# Online Purchase Intention of Fruits: Antecedents in an Integrated Model Based on Technology Acceptance Model and Perceived Risk Theory

**DOI:** 10.3389/fpsyg.2018.01521

**Published:** 2018-08-23

**Authors:** Yongchang Wei, Can Wang, Song Zhu, Hailong Xue, Fangyu Chen

**Affiliations:** ^1^School of Business Administration, Zhongnan University of Economics and Law, Wuhan, China; ^2^School of Mathematics, China University of Mining and Technology, Xuzhou, China

**Keywords:** online purchase intention, fruit e-commerce, technology acceptance model, perceived risk theory, structural equation model

## Abstract

Over recent years, online purchase platforms of fruits are increasingly emerged to advance the e-commerce development and improve quality of human life. Unfortunately, we empirically observed that a lot of enterprises selling fruits online have suffered from bankruptcy due to a lot of complicated factors, such as inefficient logistics, low acceptance of online platforms, and financial risks. One of the root causes responsible for such an unanticipated phenomenon is related to the purchase intention, which motivates us to investigate what are the dominant factors affecting the online purchase intention of fruits. The results can be of great significance to the development of fruit e-commerce enterprises in online marketing. Based on the technology acceptance model (TAM) and perceived risk theory (PRT), this research developed an integrated theoretical model to explore the influential factors underlying consumers' intention to purchase fruits online. A web-based survey of 344 consumers with ages below 30 was used to test the hypotheses in our theoretical model. Through sample collection with questionnaires, a structural equation model is developed to compute the coupling relationship between influential factors and purchase intention. The results reveal that fruit quality and price are dominantly affecting the willingness of consumers to purchase fruit. Surprisingly, we found that e-commerce platforms, information quality, and perceived risk are less significant. Finally, some specific suggestions are recommended for fruit e-commerce enterprises in devising effective marketing strategies.

## 1. Introduction

With the rapid development of mobile Internet technology, the industry of e-commerce has experienced unprecedented development due to diversified means of mobile payment. A variety of traditional industries are making efforts in capturing the business opportunities with regards to transformation and upgradation. In the wake of e-commerce development, the fruit industry is also equipped with advanced technologies, such as Internet of things (IoT), cloud computing, and blockchain technology. Purchasing fruits from online platforms to overcome the shortcomings associated with traditional channels, e.g., deteriorated fruits, high price, unsatisfied customer service, is becoming more and more popular. Over the recent years, numerous e-commerce companies are continuously built with the hope of creating value and sharing the market benefits. For example, the Fruitday, a successful leader in the fruit e-commerce, who has been upgraded from a traditional fruit supplier, gained the benefit of 500 million yuan from online in 2014 and 1 billion yuan in 2015. The customer population using mobile applications is increased to 7 million in December 2015. Unfortunately, it is surprising to observe that most Chinese companies in the field of fruit e-commerce has collectively suffered great difficulties in 2016. For example, one famous fresh products e-commerce platform, yummy77, selling high-quality organic fruits and vegetables, who ever received millions of dollars of investment form Amazon.com, was faced with serious financial crisis and applied for bankruptcy in 2016. The bankruptcy of e-commerce companies of fruits can be attributed to a lot of factors, such as the homogenization of business models, lack of cognitive differences in consumer, fruits quality and safety, website information quality. As a result, these factors lead to low acceptance of online e-commerce platforms. Consequently, two fundamental questions naturally arise: (1) what is the reason for fruit e-commerce enterprises experiencing so much difficulties? and (2) what are the dominant factors that determining the online purchase intention of fruit consumers? We contend that customer intention is one of the key factors in the development of fruit e-commerce companies, which is the focus of this research.

To the best of our knowledge, although there exists extensive research on exploring the influential factors of online purchase intention (Hausman and Siekpe, 2009; Chen et al., 2010), they are irrelevant to fruit e-commerce. For instance, Pavlou (2003) developed a model of perceptual behavior theory (PBT) and technology acceptance model (TAM) to study the impact of trust, perceived risk, perceived usefulness, perceived ease of use on purchase intention. A sample of 155 online consumers are studied and the results reveal that perceived usefulness and perceived ease of use will directly affect the transaction intention, while the trust will indirectly affect the perceived risk. Juaneda-Ayensa et al. (2016) investigated the key drivers of technology acceptance and use and their effects on purchase intention underlying omnichannel customer behavior. Tsai and Huang (2007) formulated a conceptual framework considering community-based, customization-based, desire-based, and constraint-based drivers of online customer intention. They empirically tested the data collected from a large online retailing store in Taiwan. They found that constraint-based and community-based drivers are two critical factors to retain online customers and community building dominated other factors and exceeded the combined effect of overall satisfaction and switching barriers on repurchase intentions. Chen et al. (2002) combined TAM and innovation diffusion theory in establishing a structural equation model. Finally, compatibility, perceived usefulness, and perceived ease of use are main factors that affect consumer's purchase intent. Gong et al. (2013) developed an empirical model for understanding how the demographic and media characteristics influence Chinese consumers to shop online. A nationwide online survey of 503 Chinese consumers is carried out to test the model of online purchase intention using hierarchical regression. Consumers' age, income, education and marital status, and their perceived usefulness are treated as significant inputs of online shopping intention. Lin (2007) studied the antecedents as well as effect of customer's intention to stick on a website, the model is developed and tested using a survey of 434 web users and the results confirm that the website user's willingness to stick to a website is a strong predictor of his/her intention to transact, Web managers thus need to put emphasis on the creation of the website stickiness.

In addition to the previous literature on the driving factors of online purchase, some scholars also investigated the factors affecting the purchase intention of fruits completed through traditional transaction means rather than e-commerce platforms. For example, Nandi et al. (2017) analyzed the willingness to pay for organic fruits and vegetables by using the method of conditional valuation, and analyzed 250 empirical data obtained through the consumer survey. Finally, 90% of the consumers are willing to buy high quality fruit, and family income, family size, gender and other factors will have an impact on consumer purchasing intentions. Kraus (2015) analyzed the effects of taste, quality safety and freshness on consumers' purchase intention of fruit with 200 sample. They finally concluded that fruit quality, freshness and taste are major factors affecting the behavior of consumers.

From above, we see that the vast majority of existing literature on online purchase intention and the factors affecting consumers' purchase behavior of fruits are investigated separately. It is extremely significant to combine the previous two streams of research to explore the influential factors of online purchase intention of fruits to assist decision-making for e-commerce companies. In contrast to other agricultural products, some characteristics of fruits are unique apart from that fruits can also be deteriorated easily. For example, the taste differences between different sources is usually apparent. We observe that a lot of fruit customers are very demanding on the quality of products in terms of sweetness and freshness. This motivates us to study fruit e-commerce from a perspective of consumers rather than other activities, such as production, operations, and logistics.

To identify the major factors affecting the purchase intention through Internet Applications, we designed a set of questionnaires covering multiple criteria, including fruit quality, fruit price, technical acceptance of online platforms, and perceived risks. Since online purchase activities are completed through Internet Applications, it is significant to include technical factors with regard to e-commerce platforms. In addition, risk factors should also be incorporated, because the quality of fruits is closely related to human health. For the aforementioned purposes, we developed an integrated theoretical model based on the TAM and PRT to explore the antecedents of online purchase intention. Using the data collected from representative customers, an influencing-factors model of consumers' intention to purchase fruits through Internet is further derived by structural equation model. Finally, through statistical analysis, we revealed the main factors that influencing consumers' willingness to purchase fruits. Moreover, our results are discussed in contrast to traditional fresh agricultural products, and we found that the influencing factors of fresh fruit and fresh agricultural products are different. Based on the empirical research, some useful suggestion are provided to advance the development of fruit e-commerce companies.

## 2. Theory and hypotheses

This section provides a theoretical basis for exploring the influential factors of online purchase intention of fruits. In particular, the well-recognized theoretical models, technology acceptance model and perceived risk theory, will be introduced. Based on these two theories, we will develop a conceptual framework and its related hypotheses. The conceptual framework will be leveraged to further study the influential factors through empirical research.

### 2.1. TAM and PRT

Davis (1989) proposed the technology acceptance model (TAM), which mainly uses theory of reasoned action(TRA) to study users' acceptance of information technologies. The original purpose of the proposed technology acceptance model is to explain the decisive factors that are widely accepted by the computer. The TAM posits that the individual behaviors in the use of information systems are determined by the intention, and the intention is further determined by perceived usefulness and perceived ease of use. In recent years, TAM has been successfully applied in a variety of fields to confirm its value (Ashraf et al., 2014; Escobar-Rodŕıguez and Carvajal-Trujillo, 2014; Zhang et al., 2014). It is also well recognized that it can explain the problem of consumers' purchase intention in e-commerce environment. Over the past years, online shopping has become a popular means of shopping for consumers, particularly young people, because consumers have perceived the usefulness and ease of use. This article will combine the characteristics of online shopping and fruit products, and consider the impact of various external variables, e.g., fruit quality, fruit price concessions, and website information quality, on the purchase intention of fruits consumers.

Apart from technological factors, perceived risk, which is commonly thought of as felt uncertainty regarding possible negative consequences of using a product or service (Featherman and Pavlou, 2003), is also posited as a prominent barrier to consumer acceptance of e-services. A large number of scholars have studied the perceived risk and they have their own definition of perceived risk. Peter and Ryan (1976) define perceived risk as an inhibitory effect on purchasing behavior due to the presence of expected loss of shopping or behavior. Mitchell and Taylor (1999) argued that consumers tend to reduce their perceived risk rather than maximize their perceived benefits, perceived risk is more powerful in the interpretation of consumer buying behavior. In recent years, many scholars have used the perceived risk theory (PRT)to construct the model in the study of influencing factors of consumer online shopping (Kim and Lennon, 2013; Dai et al., 2014; Nepomuceno et al., 2014). However, the lack of model lies in the complexity of the concept of perceived risk. Many problems are still controversial in this field, the perceived risk measurement model still needs to be further developed and requires empirical testing and support.

In addition to TAM and PRT, we can also use the theory of reasoned action (Mishra et al., 2014), theory of planned behavior and reasoned action (Paul et al., 2016), and e-commerce success model and commitment-trust theory (Wang et al., 2016) to study the influencing factor of consumer's intention. However, we know that fruit e-commerce has emerged as relatively new business matters, in which consumer behaviors have not yet been formed. Because you cannot see or touch the actual goods, in contrast to the traditional means of purchasing fruits, online purchase of consumers are more aware of the existence of risks. Fruits are products that cannot be tasted before they are purchased, making consumers sensitive to the perceived risk of buying fruits online. At the same time, fruits has the characteristics of freshness, perishable, fragile, and so on. These factors affect the acceptance and trust of consumers in the decision-making process.

Due to the previous reasons, this paper combines the TAM and PRT to explore what factors affect the intention to buy fruit online. Specifically. A theoretical framework will be established for explaining the intention of consumers to buy fruits online. Structural equation modeling is employed for parameters estimation and testing the proposed hypothesis. The research results will be useful for understanding the behaviors of fresh fruits e-commerce consumers and therefore promote the development of fresh fruit e-commerce.

### 2.2. Research hypotheses

The following research hypotheses are closely related to the influential factors of online consumer behavior of fruits. In addition to traditional factors in determining the intention of online purchase, our conceptual framework will be developed by incorporating the unique characteristics of fruits. Online purchase intention is a good predictor for the actual buying behavior, which is the outcome of criteria assessment of consumers regarding website quality, information search, and product evaluation (Hausman and Siekpe, 2009). Specifically, we focus on four major aspects: price concessions, fruit quality, website information quality, and perceived risks. The two factors price concessions and fruit quality are implicitly related to the key variable perceived usefulness, while the web information quality reflects the perceived ease of use in the TAM.

#### 2.2.1. Fruit price concessions

Price concession is one of the central factors that affect the purchase intention from either online or off-line for a wide range of products. Turban et al. (2002) indicates that the price is an important factor affecting the intention of customer to buy online. We contend that price is still one of the most significant factors influencing consumers' purchasing behavior. The most important reason for the rise of e-commerce is that it offers a more favorable price. At present, the fruit e-commerce is still in the infant stage and the habits of consumers have not been cultivated. The segmentation of online fruit market is far from matured. Consequently, consumers tend to be highly sensitive to price and the consumer's intention will be undoubtedly affected by price benefits. Specifically, we assume that the more price concessions the fruit e-commerce platform offers, the higher the buyer's intention to buy, which can formulated as hypothesis **H1**.

**H1:** *Fruit price concessions have a positive impact on purchase intention*.

#### 2.2.2. Fruit quality

Hughes and Merton (1996) found that the quality of fruits is very important for consumers to make purchasing decisions. Consumers are willing to pay higher price in order to obtain high-quality fruits. Huang et al. (2014) pointed out that quality certification factors have a significant impact on the behavior of consumers purchasing fresh product. Due to the virtual characteristics of e-commerce, quality and safety is a key factor in the participation of consumers in online shopping. Since fruit is one type of fresh product which deteriorate over time, the customers' requirements for quality and safety will be naturally higher than other products. In the current context of Chinese food safety problems, the quality and safety is particularly important for consumers to make purchase decisions. The relationship between fruit quality and purchase intention is characterized by hypothesis **H2**.

**H2:** *Fruit quality have a positive impact on purchase intentions*.

#### 2.2.3. Website information quality

Actually, well-designed websites and valuable information displayed may facilitates online transactions. Research has evidenced that well-developed content and functions of websites tend to increase customer satisfaction and consequently promote transactions (Hausman and Siekpe, 2009). Turban et al. (2002) argued that the integrity and accuracy of information is an important factor affecting consumers' choice of online shopping platforms. This can also be important for the development of fruit e-commerce because strong competition exists between a variety of industries. Shih (2004) found that the quality of information provided on the website has a certain impact on the behavior of consumers online shopping. During the process of commodity transaction, the information asymmetry of the parties involved is widespread in terms that consumers are often in the relatively lack of information. E-commerce facilitates the search and exchange of information than traditional business modes. This provides an opportunity for consumers to access rich information and have more options. For online retailers, the information related to the behaviors of consumers also grows rapidly, which is important in assist decision-making in terms of precision marketing. High-quality website information not only effectively attracts online consumers, but also guarantees for consumers' loyalty. Websites can provide consumers with comprehensive product information, which enable consumers to get more detailed information about fruits, which will make them more confident to buy fruits. Consequently, this study suggests that the richness of information provided by website may be an important factor in enhancing the purchase intention of consumers, as characterized by hypothesis **H3**.

**H3:** *The quality of the website information will positively affect the consumer's intention to purchase fruits*.

#### 2.2.4. Perceived risk

Perceived risks refer to the spirit cost associated with customers' purchasing behavior, which represents a kind of uncertainty about the future. This uncertainty will directly affect the consumers' purchase intention. Due to the fact that network security is highly uncertain, consumers may worry about the illegal diffusion of personal and financial information. This will possibly affect their online shopping intentions. Over the past decades, perceived risk has been attributed as an important factor affecting the acceptance of consumer online shopping, and the online shopping risk can be classified into economic risk, performance risk, psychological risk, and time risk (Forsythe and Shi, 2003; Huang et al., 2014). In this research, we include two major types of risks in the questionnaire survey: product-quality and human-health risk and information leakage risk. Following the literature, we suppose that he greater the perceived risk, the lower purchase intention, as characterized by hypothesis **H4**.

**H4:** *Perceived risk will negatively affect consumers' intention to purchase fruit*.

### 2.3. Conceptual framework

**Figure 1:**
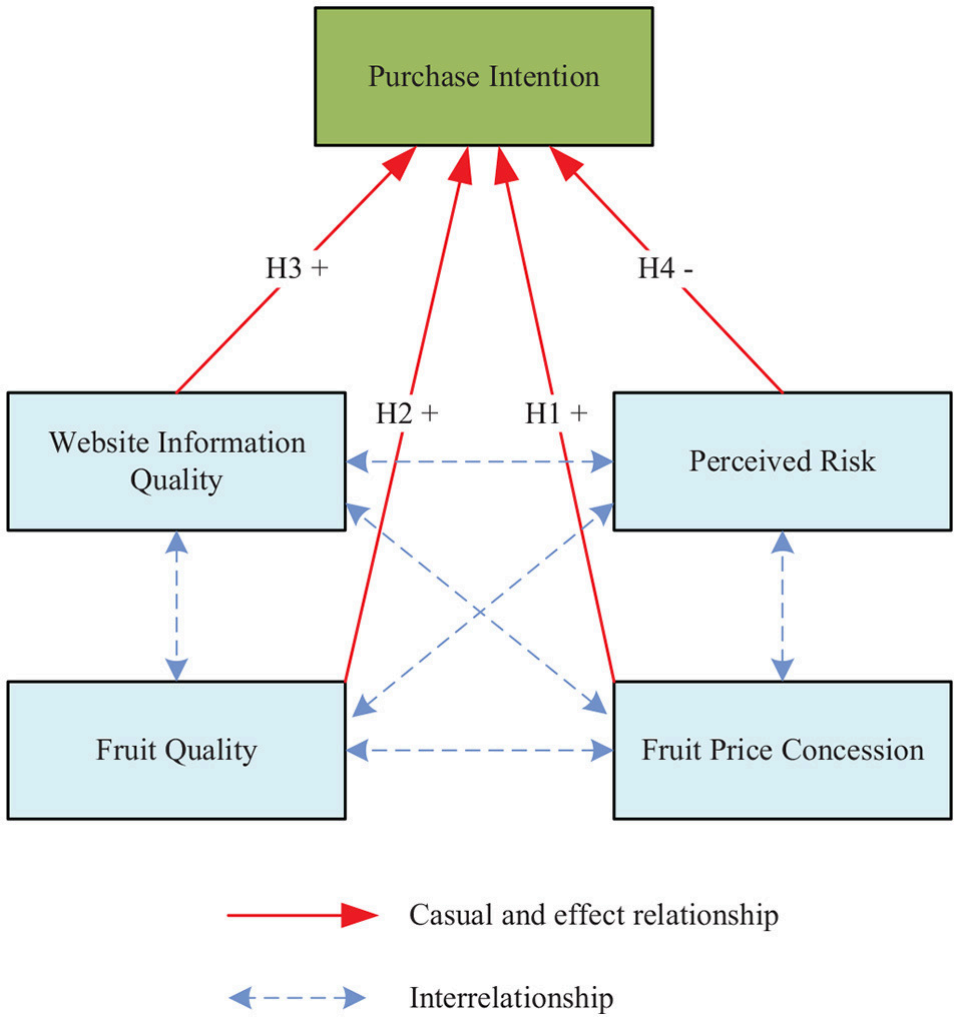
A conceptual framework for online purchase intention.

According to the above analysis and discussion, this study established a conceptual framework to exploring the online purchse intention of fruits, as shown in Figure 1. Specifically, fruit price concessions, fruit quality and quality of website information can positively affect the consumer's purchase Intention. Perceived risk may have a negative impact on the willingness to buy, the more great the perceived risk of the consumer, the lower the intention to buy. At the same time, we will also focus on the interrelationship between fruit quality, fruit price concessions, the quality of website information, and perceived risks. With data collected from representative customers, we will test the hypothesis described above by developing a structure equation model. In summary, this research aims to gain a clearer understanding of the driving factors that affect consumers' purchase intention.

## 3. Procedure and participants

Ethical approval was not required for this study in accordance with the national and institutional guidelines. Firstly, there were no unethical behaviors in the research process, because the study is focused on understanding the factors underlying consumer intention and did not involve human clinical trials or animal experiments. Secondly, the data were collected anonymously and the questionnaire was completed voluntarily. Moreover, the consent of the participants was obtained by virtue of survey completion.

All the questions are designed following the constructs in our theoretical model, as attached in the Appendix 1. Even though the literature on TAM and PRT is very rich, they are irrelevant to our problem. Therefore, by referring to the key elements in the basic TAM model (Davis, 1989), we made substantial modifications and designed all the questions following the characteristics of online fruit-purchasing. In the questionnaire survey process, we asked each participant in the questionnaire to rate the extent to which they agree with each statement by selecting a number from 1 to 7. It means that the anchor points in our questionnaire adopt the frequently used 7-level Likert-type scale, in which “1” means strongly disagree, “4” means neither agree nor disagree, and “7” means strongly agree. A total of 360 questionnaires were issued through professional websites, WeChat, and other Internet means. 359 questionnaires were actually collected, 13 invalid questionnaires were eliminated due to data incompleteness, and 344 valid questionnaires were obtained. The effective response rate of questionnaires was 95.6%.

Table 1 summarizes the basic information of the respondents. The respondents include 125 male (36.3%) and 219 female (63.7%). The possible explanation for imbalanced sex ratio is that women are more interested and enthusiastic in online shopping. According to the educational level, the respondents are mainly comprised of undergraduate and master, accounting for 81.4%. The occupations of most of the respondents are either students (60.8%) or enterprise employees (20.3%), accounting for 81.1 percent of the total sample. About 155 respondents have at least one-time experience of shopping fruits online, while 189 respondents do not have such an experience. The questionnaires of these two types of respondents are included in our further analysis, because the results on the intention of customers without purchasing experiences are also very significant in expanding the market. Actually, this study focuses on the intention rather than real transactions. In terms of age structure, the proportion of young people under the age of 30 accounts for 91.3%, which is basically consistent with the composition of Chinese Internet users. It is apparent that young people have already become the dominant consumers in e-commerce industry. In contrast to young people, older people may be risk-averse with little courage to try new things and therefore it is of less significance in investigating their purchase willingness. In the later data analysis, the data for the groups over 30 years old was thus removed.

**Table 1:** Basic information of the respondents.

**Attributes**	**Items**	**Frequency**	**Percent (%)**
Gender	Male	125	36.3
	Female	219	63.7
Age	16–20	47	13.7
	21–25	224	65.1
	26–30	43	12.5
	30–35	18	5.2
	Over 35	12	3.5
Education	Junior college	21	6.1
	College	37	10.8
	Undergraduate	183	53.2
	Master	97	28.2
	Ph.D	6	1.7
Carrer	Student	209	60.8
	Enterprise employee	70	20.3
	Government staff	11	3.2
	Staff in medical or educational institutions	14	4.1
	Other occupations	40	11.6
Purchase experience	Yes	155	45.1
	No	189	54.9

## 4. Data analysis and results

### 4.1. Measurement model and reliability test

As shown in Table 2, 15 items are included in the measurement model for testing the influential factors described previously. For instance, the website information quality is measured from three aspects: accuracy, incompleteness, and significance. The reliability of the data was analyzed by SPSS 19.0. Most of the latent variables' Cronbach's α are above 0.7 except that the Cronbach's α of the perceived risk is 0.686. The composite reliability (CR) values of all the constructs are more than 0.7. Therefore, the results in Table 2 indicate that the data has a high degree of reliability. The KMO and Bartlett's test of sphericity have also been carried out using SPSS 19.0 to obtain the KMO test value of 0.861. The Bartlett's test of sphericity is significant at the confidence level of 0.01, so the data is suitable for factor analysis.

**Table 2:** Measurement items and reliability results.

**Construct**	**Items**	**Means**	**S.D**	**Cronbach's α**	**CR**
Purchase intention	Preference in contrast to offline platforms (Q9)	4.45	1.763	0.851	0.855
	Intention strength (Q10)	3.88	1.598		
	Purchase frequency (Q11)	4.13	1.643		
Fruit quality	Overall quality (Q12)	3.89	1.666	0.870	0.874
	Freshness of fruits (Q13)	3.99	1.514		
	Fruit source can be inquired (Q14)	3.87	1.564		
Fruit price concessions	Price comparison between online and offline (Q15)	4.19	1.917	0.803	0.803
	Price promotions (Q16)	4.63	1.826		
	Price/Performance ratio (Q17)	4.13	1.669		
Website information quality	Information accuracy (Q18)	4.40	1.493	0.855	0.859
	Information incompleteness (Q19)	4.28	1.461		
	Information significance (Q20)	4.44	1.468		
Perceived risk	Worry about the freshness (Q21)	5.18	1.504	0.686	0.712
	Worry about the residuals of hazard materials (Q22)	4.92	1.559		
	Worry about the risk of information privacy (Q23)	4.75	1.908		

Using principal component analysis, the data was analyzed and processed by the maximum variance rotation. It is expected that the total variation can be explained to the greatest extent with the least common factors. The results are presented in Table 3, from which five factors are extracted with eigenvalues greater than 1. For clarity, the coefficients less than 0.3 are not displayed. These five factors correspond to the latent variables as shown in the conceptual framework. As shown in Table 4 their eigenvalue accumulations explained the overall variance of 69.09%. Therefore, we conclude that our data is reliable and can be further analyzed with SEM.

**Table 3:** Principal component analysis with varimax rotations.

**Items**	**Factor 1**	**Factor 2**	**Factor 3**	**Factor 4**	**Factor 5**
Q9	0.821	–	–	–	–
Q10	0.82	–	–	–	–
Q11	0.787	–	–	–	–
Q12	–	–	–	–	0.416
Q13	–	–	–	–	0.425
Q14	0.528	–	–	–	0.58
Q15	–	–	0.864	–	–
Q16	0.325	–	0.71	–	–
Q17	0.311	–	0.759	–	–
Q18	–	0.805	–	–	–
Q19	–	0.855	–	–	–
Q20	–	0.793	–	–	–
Q21	–	–	–	0.883	–
Q22	–	–	–	0.855	–
Q23	–	–	–	0.922	–

Coefficients less than 0.3 are marked with “−.”

**Table 4:** Total variances explained.

	**Initial eigenvalues**			**Extraction sums of squared loadings**			**Rotation sums of squared loadings**		
**Component**	**Total**	**% of Variance**	**Cumulative %**	**Total**	**% of Variance**	**Cumulative %**	**Total**	**% of Variance**	**Cumulative %**
1	9.103	41.375	41.375	9.103	41.375	41.375	4.509	20.496	20.496
2	2.228	10.128	51.503	2.228	10.128	51.503	4.184	19.019	39.515
3	1.482	6.738	58.241	1.482	6.738	58.241	2.421	11.006	50.521
4	1.309	5.948	64.189	1.309	5.948	64.189	2.181	9.915	60.436
5	1.078	4.901	69.09	1.078	4.901	69.09	1.904	8.655	69.09

From the above analysis, we know that the measurable variables are capable of interpreting latent variables. Here we focus on analyzing the correlation between the latent variables to provide a foundation for exploring the causal relationship in the structural model. The casual relationship between the latent variables is meaningful if the correlation is strong. As shown in Table 5, we can see that the correlational relationship between purchase intention and perceived risk is relatively weak, while the correlation coefficients between purchase intention and other latent variables are at least greater than 0.484. The fruit quality is also closely related to fruit price concessions and the website information quality, and the variable of fruit price concessions is also positively correlated to website information quality. Actually, as can be seen later, these quantitative results are highly consistent with the results presented with the SEM technique.

**Table 5:** Correlation analysis.

**Variable**	**Mean**	**S.D**.	**1**	**2**	**3**	**4**	**5**
Purchase intention	4.154	1.286	1				
Fruit quality	3.919	1.254	0.669^**^	1			
Fruit price concessions	4.316	1.293	0.530^**^	0.496^**^	1		
Website information quality	4.374	1.143	0.468^**^	0.600^**^	0.474^**^	1	
Perceived risk	4.949	1.008	−0.055	-0.107	0.1	0.045	1

The symbol

** *indicates that the correlation is significant at the 0.01 level*.

### 4.2. Structural model

**Figure 2:**
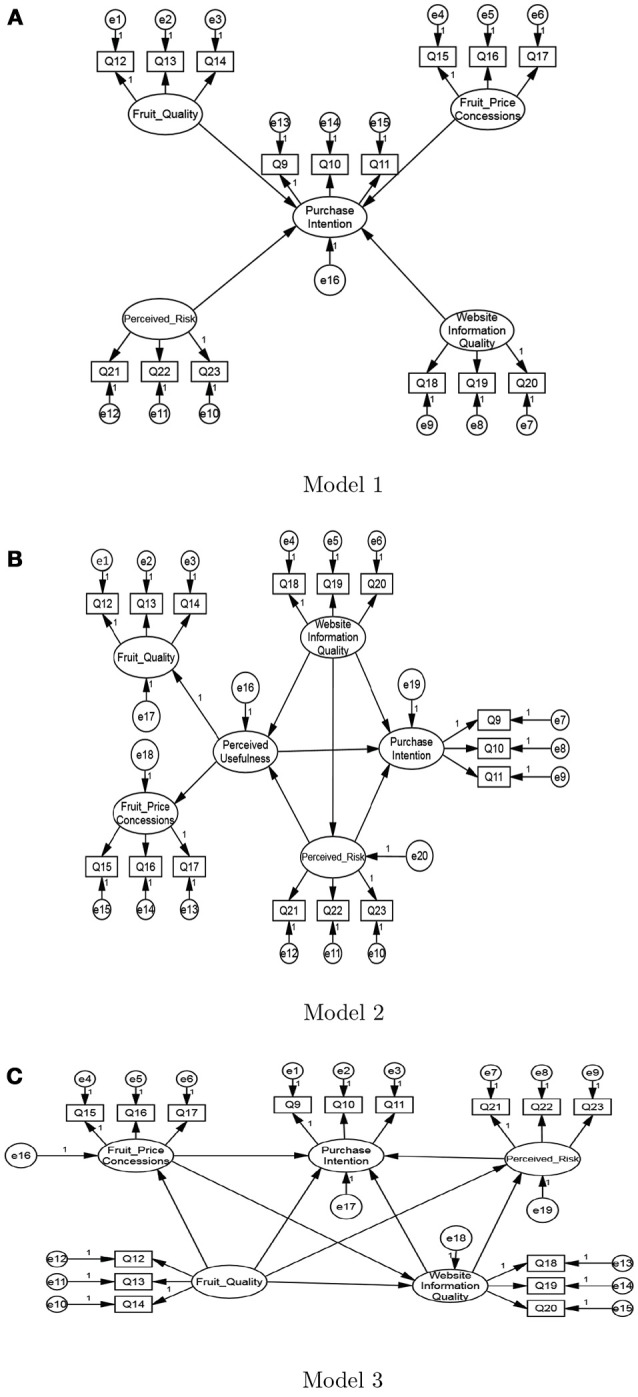
The three candidate structural models for comparison.

The causal and effect structure of the proposed conceptual framework was tested using structure equation modeling (SEM). Three SEM model structures are deliberately designed for comparison purpose, as shown in Figure 2. The main differences between the three models lie in the structure complexity. In Model 1, we only incorporate the casual effects between the purchase intention and other latent variables. In Model 2, we explicitly include the perceived usefulness variable to follow the traditional TAM. However, we don't include the perceived ease of use since it is already reflected by the website information quality. In Model 3, we further incorporate the casual relationships between different latent variables into our model. Not that we eliminated the perceived usefulness variable for simplicity and actually we found that a second-order model, as represented in Model 2, does not substantially improve the model fitness.

The following metrics were used to assess the fitness of SEM: chi-square /degrees of freedom (χ^2^/*df*), goodness-of-fit index (GFI), adjusted goodness-of-fit index index (AGFI), normed fit index (NFI), comparative fit index (CFI), and root mean square of approximation (RMSEA). According to Scott (1995), the χ^2^/*df* of a fit model should be less than 3.0, CFI, GFI and NFI should be greater than 0.9, and AGFI should be greater than 0.8. The root mean square error of approximation (RMSEA) should be below the recommended range of acceptability (0.05–0.08) recommended by MacCallum et al. (2006).

**Figure 3:**
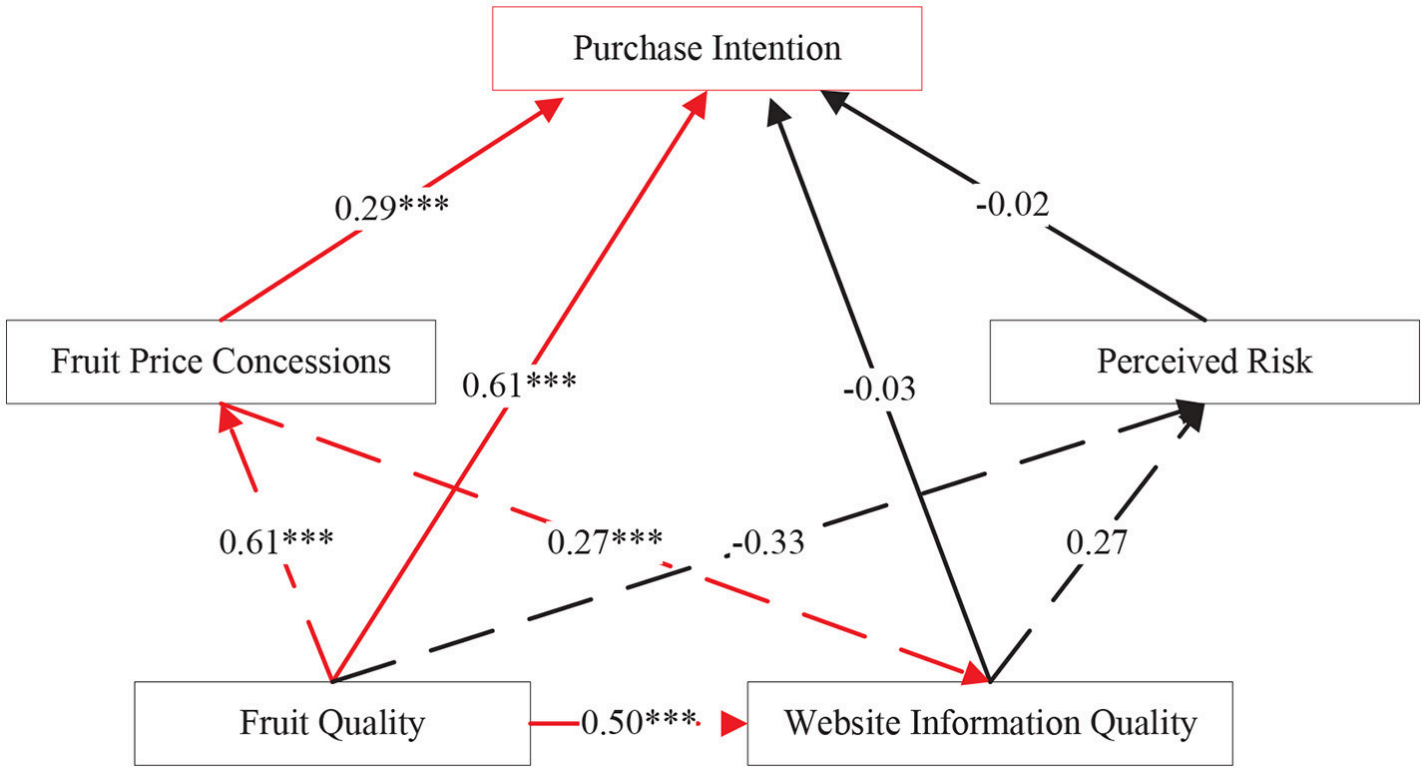
Standardized parameter estimation results of Model 3 (***p < 0.001).

The results on the fitting indexes of the three models are shown in Table 6. By comparison, we found that the performance of Model 1 is relatively poor, and the fitting indexes of Model 2 and Model 3 satisfy the aforementioned standards (Scott, 1995; MacCallum et al., 2006). Through further comparison between Model 2 and Model 3, we found that the fitting performance of Model 3 is better than Model 2. Therefore, we conclude that Model 3 can best fit our problem, the results of which are presented in Figure 3 and Table 7.

**Table 6:** The fitting results of the three models.

**Index**	**Items**	**Model 1**	**Model 2**	**Model 3**	**Standard**
Absolute fitting index	χ^2^	453.7	202.684	196.439	–
	*df*	86	82	81	–
	χ^2^/*df*	5.276	2.472	2.425	< 3
	RMSEA	0.117	0.069	0.067	< 0.08
	GFI	0.844	0.923	0.925	>0.9
Relative fitting index	NFI	0.82	0.92	0.922	>0.9
	CFI	0.848	0.95	0.952	>0.9
	IFI	0.849	0.951	0.953	>0.9
	RFI	0.78	0.897	0.9	>0.9
Simple fitting index	PNFI	0.672	0.718	0.711	>0.5
	PGFI	0.605	0.631	0.625	>0.5

**Table 7:** The estimation results between variables in Model 3.

**Effect**	**Cause**	**UNSTD**	**S.E**.	**C.R**.	***p* value**	**STD**	**SEM**	**1-SEM**
Fruit price concessions	Fruit quality	0.632	0.076	8.268	***	0.614	0.377	0.623
Website information quality	Fruit quality	0.524	0.081	6.453	***	0.496	0.246	0.754
Website information quality	Fruit price concessions	0.276	0.077	3.585	***	0.269	0.072	0.928
Perceived risk	Fruit quality	−0.333	0.103	−3.239	0.001	−0.331	0.11	0.89
Perceived risk	Website information quality	0.256	0.096	2.663	0.008	0.27	0.073	0.927
Purchase intention	Fruit price concessions	0.309	0.075	4.089	***	0.293	0.086	0.914
Purchase intention	Perceived risk	−0.02	0.055	−0.371	0.711	−0.019	0	1
Purchase intention	Fruit quality	0.665	0.094	7.052	***	0.612	0.375	0.625
Purchase intention	Website information quality	−0.033	0.074	−0.438	0.661	−0.032	0.001	0.999
Q9	Purchase intention	1				0.767	0.588	0.412
Q10	Purchase intention	1.094	0.07	15.595	***	0.881	0.776	0.224
Q11	Purchase intention	0.996	0.07	14.162	***	0.791	0.626	0.374
Q15	Fruit price concessions	1				0.697	0.486	0.514
Q16	Fruit price concessions	1.025	0.092	11.19	***	0.732	0.536	0.464
Q17	Fruit price concessions	1.127	0.093	12.086	***	0.843	0.711	0.289
Q21	Perceived risk	1				0.768	0.59	0.41
Q22	Perceived risk	1.072	0.15	7.141	***	0.809	0.654	0.346
Q23	Perceived risk	0.594	0.098	6.044	***	0.405	0.164	0.836
Q14	Fruit quality	1				0.749	0.561	0.439
Q13	Fruit quality	1.166	0.074	15.792	***	0.888	0.789	0.211
Q12	Fruit quality	1.19	0.077	15.426	***	0.864	0.746	0.254
Q18	Website information quality	1				0.81	0.656	0.344
Q19	Website information quality	1.073	0.066	16.33	***	0.879	0.773	0.227
Q20	Website information quality	0.934	0.065	14.313	***	0.764	0.584	0.416

In Table 7, *UNSTD* is the un-standardized estimates and *STD* is the standardized estimates. Generally, we see that the standardized parameter estimations between the variables fall inside the interval [0.38, 0.98]. The statistical significance is significant at the level of 0.001, indicating that the indicators have a strong ability to explain Model 3. Based on the previous results, we can draw the the following managerial insights from two aspects: the driving factors of purchase intention; the interrelationship between these factors.

(1) From Table 7, we see that the CR values of the hypotheses “website information quality has a positive effect on the purchase intention” and “perceived risk has a negative effect on the purchase intention” are less than the reference value of 1.96 and the *p* values do not reach the critical significance level 0.05. It means that those two hypotheses are not true, while other two hypotheses hold significantly. The results can be further explained as follows. Firstly, we know that price may be a main factor underlying purchase intention for numerous products. Thus, we can draw the conclusion that fruit price concessions affects the consumer purchase intention, which coincides with the characteristics of fruit e-commerce. In particular, the more favorable the price of fruits, the consumer will be more willing to purchase. Secondly, we reveal that fruit quality is the most important driving factor for fruits. As e-commerce is inherently virtual, consumers are more likely to pay attention to the quality of fruits. Thus, it is natural that fruit quality has a positive impact on consumer purchase intention in terms that the better fruit quality, the purchase intention of consumers will be stronger. Thirdly, the quality of information on the website has no impact on consumers' purchasing intention. This indicates that consumers' intention to purchase fruit is irrelevant to their expectation of the quality of the site's information. This result may be attributed to the fact that consumers' expectation on the quality of the website information can be readily satisfied with the advancement of information technologies. Finally, it is revealed that perceived risk also has no significant effect on consumer purchasing intention. This result is counter-intuitive. We contend the perceived risk with regards to payment security and personal information privacy can be solved through technical solutions with matured approaches. Actually, the current online payment is highly contingent upon third-party platforms, such as Alipay and WeChat. The payment security can be guaranteed for fruit e-commerce transactions and the related risks can be reduced without attracting consumers' attention.

(2) Regarding the interdependency among influential factors, we found that the effect of fruit quality on price concessions and website information quality is significant at level of 0.001, implying that fruit quality significantly affects fruit price concessions and website information quality. In addition, the effect of fruit price concessions on website information quality is significant at level of 0.001, implying that fruit price concessions significantly affects website information quality. The higher the quality of fruits and the better the quality of information, consumers will be more comfortable in buying fruits on the website. This will indirectly improve the purchase intention. Secondly, the quality of fruit will also have a positive impact on fruit prices concessions. The higher the quality of the fruit, the price of the fruit will be increased and unit profits of fruit will be reduced. In order to gain more profits, fruit e-commerce enterprises will use promotion activities, such as price concessions and advertisement, to increase the selling amount of fruits. This will not only increases the profits of fruit e-commerce, but also indirectly improves the purchase intention. Finally, fruit price concessions also have a positive impact on the quality of website information, the more fruit price concessions, the greater the dependence of consumers on the site, the purchase intention via websites will be stronger.

## 5. Conclusion and discussion

The development of fruit e-commerce substantially improve the quality of human life. From a perspective of consumer behaviors, this paper developed an empirical model for exploring the influential factors of online purchase intention for fruits. The results obtained would provide insightful support for the development of fruit e-commerce.

Through empirical study, we found that fruit price concessions significantly affect consumers' purchase intention. The most important reason for the rise of e-commerce is that it provides a more favorable price. At present, the fruit e-commerce is still in the initial stage, consumption habits has not been cultivated completely and it is not surprising that consumers are very sensitive to price. We also found that fruit quality significantly affects consumers' purchase intention. The estimated parameter on the impact of fruit quality on consumers' purchase intention is 0.61, implying that fruit quality is the most important factor affecting consumers online shopping. The better quality of fruits that e-commerce platforms provide, the stronger the intention of consumers to buy. There are many problems on Chinese food safety, while online shopping faces more risks than traditional purchase channels and therefore customers may be more cautious on fruit quality in online shopping. The perceived gap between the actual fruit quality and its expectations is an important factor in hindering consumers from buying fruit online.

In this research, we show that the quality of website information has no significant effect on consumer's purchasing intention. Shih (2004) found that the quality of information from website has a certain impact on the behavior of consumers' online shopping. The reason is that the quality of the information from many websites is generally low, the information displayed on websites is inconsistent with the real information of the fruits, and generally the information from websites is not updated timely. Finally, we demonstrate that the perceived risk has no significant effect on consumer's purchasing intention. This suggests that consumers' intention is irrelevant to their perceived risk. The study in Cheung et al. (2005) found that increased trust in the payment environment was an effective way of promoting online sales. This is different from our conclusion, the reason may be that the perceived risks from aspects such as payment security and personal information privacy can be effectively settled by the increasingly matured third-party payment ways.

Based on our research results, the following useful suggestions to fruit e-commerce enterprises are put forward. First, we should emphasize on product quality. According to the characteristics of fresh fruits, the whole process from the production process to the consumption throughout a supply chain should be controlled strictly ensuring that consumers can retrieve fresh and healthy fruits. Second, price promotions can be leveraged to increase the sale amount of fruits. Low-price competition may be an important reason for the difficulties of fruit e-commerce companies faced currently. Even if low-price can attract consumers in short-term, but the price recovery in the long term can easily lead to customer loss. Therefore, price concessions should be employed in a stable way rather than attracting customers for short-term benefits. Third, although the quality of information seems to be insignificant in affecting the purchase intention of consumers, but we still should optimize the elements in the websites for ease of use because the development of fruit e-commerce are still in the infant stage. We must point out the limitations in this paper. We only consider the influential factors from the perspective of consumer behaviors while ignored other significant factors, such as financial factor and operational factors concerned in the field of logistics and supply chain management. These factors are also significant which deserve future research.

## Author contributions

YW and FC developed the conceptual framework and revised the whole paper. CW analyzed the data and written the main sections. HX collected the data and discussed the results. SZ made substantial contributions in analyzed the data in the revision process.

### Conflict of interest statement

The authors declare that the research was conducted in the absence of any commercial or financial relationships that could be construed as a potential conflict of interest.

## References

[B1] Ashraf A. R. Thongpapanl N. Auh S. (2014). The application of the technology acceptance model under different cultural contexts: the case of online shopping adoption. J. Int. Market. 22, 68–93. 10.1509/jim.14.0065

[B9] Chen L. Gillenson M. L. Sherrell D. L. (2002). Enticing online consumers: an extended technology acceptance perspective. Inform. Manage. 39, 705–719. 10.1016/S0378-7206(01)00127-6

[B2] Chen Y.-H. Hsu I.-C. Lin C.-C. (2010). Website attributes that increase consumer purchase intention: a conjoint analysis. J. Business Res. 63, 1007–1014. 10.1016/j.jbusres.2009.01.023

[B3] Cheung C. M. Chan G. W. Limayem M. (2005). A critical review of online consumer behavior: empirical research. J. Electr. Commerce Organ. 3:1 10.4018/jeco.2005100101

[B4] Dai B. Forsythe S. Kwon W.-S. (2014). The impact of online shopping experience on risk perceptions and online purchase intentions: does product category matter? *J. Electr. Commerce Res*. 15:13. Available Online at: http://www.jecr.org/sites/default/files/15_1_p02.pdf

[B5] Davis F. D. (1989). Perceived usefulness, perceived ease of use, and user acceptance of information technology. MIS Q. 13, 319–340. 10.2307/249008

[B6] Escobar-Rodríguez T. Carvajal-Trujillo E. (2014). Online purchasing tickets for low cost carriers: an application of the unified theory of acceptance and use of technology (utaut) model. Tourism Manage. 43, 70–88. 10.1016/j.tourman.2014.01.017

[B7] Featherman M. S. Pavlou P. A. (2003). Predicting e-services adoption: a perceived risk facets perspective. Int. J. Hum. Comput. Stud. 59, 451–474. 10.1016/S1071-5819(03)00111-3

[B8] Forsythe S. M. Shi B. (2003). Consumer patronage and risk perceptions in internet shopping. J. Busin. Res. 56, 867–875. 10.1016/S0148-2963(01)00273-9

[B10] Gong W. Stump R. L. Maddox L. M. (2013). Factors influencing consumers' online shopping in china. J. Asia Busin. Stud. 7, 214–230. 10.1108/JABS-02-2013-0006

[B11] Hausman A. V. Siekpe J. S. (2009). The effect of web interface features on consumer online purchase intentions. J. Busin. Res. 62, 5–13. 10.1016/j.jbusres.2008.01.018

[B12] Huang L. Feng J. Yan F. (2014). Study on the perceived risk about the online shopping for fresh agricultural commodities and customer acquisition. Asian Agricult. Res. 6:1. Available Online at: https://EconPapers.repec.org/RePEc:ags:asagre:180422

[B13] Hughes D. Merton I. (1996). “partnership in produce”: the j sainsbury approach to managing the fresh produce supply chain. Supply Chain Manage. Int. J. 1, 4–6. 10.1108/13598549610155251

[B14] Juaneda-Ayensa E. Mosquera A. Sierra Murillo Y. (2016). Omnichannel customer behavior: key drivers of technology acceptance and use and their effects on purchase intention. Front. Psychol. 7:1117. 10.3389/fpsyg.2016.01117 27516749 PMC4963459

[B15] Kim J. Lennon S. J. (2013). Effects of reputation and website quality on online consumers' emotion, perceived risk and purchase intention: based on the stimulus-organism-response model. J. Res. Interact. Market. 7, 33–56. 10.1108/17505931311316734

[B16] Kraus A. (2015). Factors influencing the decisions to buy and consume functional food. Brit. Food J. 117, 1622–1636. 10.1108/BFJ-08-2014-0301

[B17] Lin J. C.-C. (2007). Online stickiness: its antecedents and effect on purchasing intention. Behav. Inform. Technol. 26, 507–516. 10.1080/01449290600740843

[B18] MacCallum R. C. Browne M. W. Cai L. (2006). Testing differences between nested covariance structure models: power analysis and null hypotheses. Psychol. Methods 11:19. 10.1037/1082-989X.11.1.19 16594765

[B19] Mishra D. Akman I. Mishra A. (2014). Theory of reasoned action application for green information technology acceptance. Comput. Hum. Behav. 36, 29–40. 10.1016/j.chb.2014.03.030

[B20] Mitchell P. Taylor L. M. (1999). Shape constancy and theory of mind: is there a link? Cognition 70, 167–190. 10.1016/S0010-0277(99)00011-6 10349762

[B21] Nandi R. Bokelmann W. Gowdru N. V. Dias G. (2017). Factors influencing consumers' willingness to pay for organic fruits and vegetables: empirical evidence from a consumer survey in india. J. Food Prod. Market. 23, 430–451. 10.1080/10454446.2015.1048018

[B22] Nepomuceno M. V. Laroche M. Richard M.-O. (2014). How to reduce perceived risk when buying online: the interactions between intangibility, product knowledge, brand familiarity, privacy and security concerns. J. Retail. Consum. Serv. 21, 619–629. 10.1016/j.jretconser.2013.11.006

[B23] Paul J. Modi A. Patel J. (2016). Predicting green product consumption using theory of planned behavior and reasoned action. J. Retail. Consum. Serv. 29, 123–134. 10.1016/j.jretconser.2015.11.006

[B24] Pavlou P. A. (2003). Consumer acceptance of electronic commerce: integrating trust and risk with the technology acceptance model. Int. J. Electr. Commerce 7, 101–134. 10.1080/10864415.2003.11044275

[B25] Peter J. P. Ryan M. J. (1976). An investigation of perceived risk at the brand level. J. Market. Res. 13, 184–188. 10.2307/3150856

[B26] Scott J. E. (1995). The measurement of information systems effectiveness: evaluating a measuring instrument. ACM SIGMIS Database 26, 43–61. 10.1145/206476.206484

[B27] Shih H.-P. (2004). An empirical study on predicting user acceptance of e-shopping on the web. Inform. Manag. 41, 351–368. 10.1016/S0378-7206(03)00079-X

[B28] Tsai H.-T. Huang H.-C. (2007). Determinants of e-repurchase intentions: an integrative model of quadruple retention drivers. Inform. Manag. 44, 231–239. 10.1016/j.im.2006.11.006

[B29] Turban E. King D. Lee J. Viehland D. (2002). Electronic commerce: a managerial perspective 2002. Prentice Hall 13:4.

[B30] Wang W.-T. Wang Y.-S. Liu E.-R. (2016). The stickiness intention of group-buying websites: the integration of the commitment–trust theory and e-commerce success model. Inform. Manage. 53, 625–642. 10.1016/j.im.2016.01.006

[B31] Zhang K. Z. Zhao S. J. Cheung C. M. Lee M. K. (2014). Examining the influence of online reviews on consumers' decision-making: a heuristic–systematic model. Decis. Supp. Syst. 67, 78–89. 10.1016/j.dss.2014.08.005

