# Supplementary material for: Online Purchase Intention of Fruits: Antecedents in an Integrated Model Based on Technology Acceptance Model and Perceived Risk Theory
Source: Front Psychol. 2018 Aug 23;9:1521. doi: 10.3389/fpsyg.2018.01521 (PMC6116833; doi:10.3389/fpsyg.2018.01521)
Supplement: Supplementary file 1 [file Data_Sheet_1.docx]

**Appendix 1: Questionnaire on the factors influencing consumers' online purchasing intention**

**Part 1: Basic information**

Please take a few minutes to let us know you. (Circle only ONE answer for the following questions.)

1. What is your age?

A.16-20 B.21-25 C.26-30 D.31-35 E. Over 35

2. What is your academic qualification?

A. Junior college B. College C. Undergraduate D. Master E. Ph.D

3. What is your gender?

A. Male B. Female

4. What is your career?

A. Student B. Enterprise employee C. Government staff D. Staff in medical or educational institutions E. Other occupations

5. How much do you live on a monthly basis, if you are a student?

A. Under 800 B. 800-1200 C. 1200-1600 D.1600-2000 E. Over 2000

6. What is your monthly salary, if you are working?

A. Under 3000 B.3000-6000 C.6000-10000 D.10000-15000 E. Over 15000

7. Have you ever purchased fruits on the fruit e-commerce platform?

A. Yes B. No

8. Which e-commerce platforms have you purchased fruits on? ____________________________

**Part 2: Variable measurement**

Circle only one answer for the following questions, in which the number indicates the extent to which they agree with the statement. For example, “1” means strongly disagree, “4” means neither agree nor disagree, and “7” means strongly agree.

**Table A1. Questions for variable measurement**

|  | Strongly  Disagree | → | | | | | Strongly  Agree |
| --- | --- | --- | --- | --- | --- | --- | --- |
|  | 1 | 2 | 3 | 4 | 5 | 6 | 7 |
|  | 1 | 2 | 3 | 4 | 5 | 6 | 7 |
| 9. I would like to buy fruits on the fruit e-commerce platform. | 1 | 2 | 3 | 4 | 5 | 6 | 7 |
| 10.I have a strong intention to buy fruits on the fruit e-commerce platform. | 1 | 2 | 3 | 4 | 5 | 6 | 7 |
| 11. I will frequently buy fruits from the fruit e-commerce platform in the future. | 1 | 2 | 3 | 4 | 5 | 6 | 7 |
| 12. The fruit e-commerce platform provides me with high-quality fruits. | 1 | 2 | 3 | 4 | 5 | 6 | 7 |
| 13. The fruit e-commerce platform provides me with fresh and good-taste fruits. | 1 | 2 | 3 | 4 | 5 | 6 | 7 |
| 14. The fruit e-commerce platform provides me with regular and clear sources of fruits . | 1 | 2 | 3 | 4 | 5 | 6 | 7 |
| 15. I think the price of fruits on the fruit e-commerce platform is cheaper than offline. | 1 | 2 | 3 | 4 | 5 | 6 | 7 |
| 16. I think the price of fruit e-commerce is relatively favorable, which will attract me to buy it. | 1 | 2 | 3 | 4 | 5 | 6 | 7 |
| 17. I think the fruit e-commerce platform provides more cost-effective fruits compared with offline. | 1 | 2 | 3 | 4 | 5 | 6 | 7 |
| 18. The fruit e-commerce platform will provide me with accurate information. | 1 | 2 | 3 | 4 | 5 | 6 | 7 |
| 19. Fruit e-commerce platform will provide me with detailed and complete fruit information. | 1 | 2 | 3 | 4 | 5 | 6 | 7 |
| 20. I can collect the fruit information I needed on the fruit e-commerce platform. | 1 | 2 | 3 | 4 | 5 | 6 | 7 |
| 21. I worry about that the fruits on the fruit e-commerce platform are not fresh. | 1 | 2 | 3 | 4 | 5 | 6 | 7 |
| 22. I worry about the presence of harmful substances in the fruits on the fruit e-commerce platform. | 1 | 2 | 3 | 4 | 5 | 6 | 7 |
| 23. I worry about the disclosure risk of personal information on the fruit e-commerce platform. | 1 | 2 | 3 | 4 | 5 | 6 | 7 |
